# ASO Author Reflections: Preventable Mortality Following Esophago-Gastric Cancer Resection

**DOI:** 10.1245/s10434-023-13585-2

**Published:** 2023-05-08

**Authors:** David S. Liu, Tim Bright, David I. Watson

**Affiliations:** 1 Upper Gastrointestinal Surgery Unit, Division of Surgery, Anaesthesia and Procedural Medicine, Austin Hospital, Heidelberg, VIC Australia; 2 Division of Cancer Surgery, Peter MacCallum Cancer Centre, Melbourne, VIC Australia; 3 General and Gastrointestinal Surgery Research and Trials Group, The University of Melbourne Department of Surgery, Austin Health, Heidelberg, VIC Australia; 4 Flinders Medical Centre, Oesophago-gastric Surgery Unit, Bedford Park, SA Australia; 5 Discipline of Surgery, College of Medicine and Public Health, Flinders University, Bedford Park, SA Australia; 6 Division of Surgery, Anaesthesia and Procedural Medicine, Austin Hospital, Heidelberg, VIC Australia

## Past

Despite advances in surgical and perioperative care, esophago-gastric cancer surgery is still associated with relatively high perioperative mortality. Recent international studies suggest that this rate is as high as 5%, particularly from lower surgical volume centers.^1^ From an epidemiological perspective, many studies derived from cancer registries and administrative databases have investigated postoperative mortality and its associated risk factors.^2,3^ However, few have quantified *preventable* postoperative mortality or attempted to unravel its underlying etiologies at an individual level. Importantly, such analyses are needed to better direct quality-improvement efforts and inform system processes to improve patient care in the future.

The Australian and New Zealand Audit of Surgical Mortality (ANZASM) collects data on all in-hospital surgical mortality across Australia. This program is managed by the Royal Australasian College of Surgeons and includes an independent peer review process for all cases.^4^ This dataset affords the opportunity to: (1) determine the causes of mortality following esophago-gastric cancer resections in Australia, (2) quantify the proportion of potentially preventable deaths, and (3) describe the underlying factors and clinical management issues that contributed to preventable mortality.

## Present

In this study using ANZASM data,^5^ we analyzed all in-hospital mortalities following esophago-gastric cancer surgery in Australia over 10 years. We identified 636 complications and 123 clinical management issues in 105 mortalities. The most common causes of death were cardio-respiratory in nature. Based on independent reviewers’ assessment of each case, we report that 47% of deaths were deemed potentially preventable.

After comparing potentially preventable and non-preventable deaths, we found that potentially preventable cases were characterized by higher rates of generalized sepsis, multiorgan dysfunction syndrome, reoperation, small bowel obstruction, delayed conduit emptying that required reintervention, and jejunostomy-related issues. Importantly, potentially preventable mortalities had a significantly higher number of clinical management issues per patient, which adversely impacted on all phases of patient care. Moreover, unlike perioperative mortality in general, preventable mortality was not predictable preoperatively.

Our thematic analysis of clinical management issues has highlighted recurrent areas of deficiency in patient care. Preoperatively, inadequate surgical assessment, planning, and/or optimization of patient fitness for surgery, inappropriate decision to offer surgery, and incorrect choice or approach to an operation were the most common preventable deficiencies. Intraoperatively, technical error, incorrect decision-making, and lack of senior surgeon availability were common preventable contributors to mortality. Postoperatively, failure to rescue, poor decision-making, and treatment delays were the most common themes identified that were potentially preventable and contributed to an inability to salvage patients when complications occurred.

## Future

The proportion of potentially preventable mortalities was surprisingly high in our cohort. This requires validation in other cohorts. In this way, our study encourages other disciplines and hospitals globally to conduct similar analyses of mortality data. Moreover, our findings can be used as a basis for prioritizing quality improvement initiatives within each phase of patient care.

Overall, whilst it is generally accepted that esophago-gastric cancer surgery carries significant morbidity risks, ultimately the onus is on us as surgical leaders to implement processes to safeguard against omissions and commissions, however small, to avoid the conversion of morbidity into mortality.
